# Influence of Selected Indicators of Healthcare System Functioning Evaluation on the Health Result

**DOI:** 10.3390/ijerph192114618

**Published:** 2022-11-07

**Authors:** Olga Partyka, Monika Pajewska, Aleksandra Czerw, Katarzyna Sygit, Kamila Kmieć, Oleh Lyubinets, Mateusz Niemiec, Mateusz Kaczmarski, Izabela Gąska, Grzegorz Juszczyk, Edyta Krzych-Fałta, Tomasz Banaś, Dariusz A. Kosior, Andrzej Deptała, Artur Kotwas, Ewa Bandurska, Weronika Ciećko, Elżbieta Cipora

**Affiliations:** 1 Department of Health Economics and Medical Law, Medical University of Warsaw, 01-445 Warsaw, Poland; 2 Department of Economic and System Analyses, National Institute of Public Health NIH-National Research Institute, 00-791 Warsaw, Poland; 3 Faculty of Health Sciences, Calisia University, 62-800 Kalisz, Poland; 4 Department of Public Health, Danylo Halytsky Lviv National Medical University, 79010 Lviv, Ukraine; 5 Medical Institute, Jan Grodek State University in Sanok, 38-500 Sanok, Poland; 6 Department of Public Health, Medical University of Warsaw, 02-091 Warsaw, Poland; 7 Department of Basic of Nursing, Faculty of Health Sciences, Medical University of Warsaw, 01-445 Warsaw, Poland; 8 Department of Gynaecology and Oncology, Jagiellonian University Medical College, 31-501 Cracow, Poland; 9 Department of Radiotherapy, Maria Sklodowska-Curie Institute-Oncology Centre, 31-115 Cracow, Poland; 10 Clinical and Research Department of Applied Physiology, Mossakowski Medical Research Institute Polis Academy of Sciences, 02-106 Warsaw, Poland; 11 Department of Cardiology and Hypertension with Electrophysiological Lab, Central Research Hospital of the Ministry of the Interior and Administration, 02-507 Warsaw, Poland; 12 Department of Cancer Prevention, Medical University of Warsaw, 02-091 Warsaw, Poland; 13 Subdepartment of Social Medicine and Public Health, Department of Social Medicine, Pomeranian Medical University in Szczecin, 71-210 Szczecin, Poland; 14 Center for Competence Development, Integrated Care and e-Health, Medical University of Gdańsk, 80-204 Gdansk, Poland

**Keywords:** healthcare indicators, public health, healthcare public health

## Abstract

Background: According to the World Health Organization’s statistics, 7 of the 10 main causes of death in 2019 were noncommunicable diseases. Health indicators are measures used to evaluate public health system effectiveness and functioning. Monitoring mortality rates from leading causes, life expectancy and other health indicators is essential to address their causes and adapt health systems to react adequately. The aim of this study is to present the dependencies of selected health care indicators and health outcomes. Methods: Based on the literature review conducted, selected health indicators, along with healthcare system data, were analyzed using Pearson’s r correlation. The analyses included data from the Organization for Economic Cooperation and Development (OECD) presented in statistics and the Health at a Glance 2021 report and data collected as part of the preparation of the Financing Global Health 2020 report by the Institute for Health Metrics and Evaluation. Results: Health system resources are linked to health outcomes. The number of medical consultations, the number of nurses per patient or the level of financing of services under general health insurance are related to life expectancy and deaths due to causes that could have been avoided or treated. Conclusions: Life expectancy is positively correlated with access to general health insurance and public expenditure on healthcare. There is a need for all countries to provide their citizens with broad access to healthcare services.

## 1. Introduction

According to the World Health Organization’s (WHO) Global Health Estimates, the main causes of death are cardiovascular diseases (among which ischemic heart disease and stroke are leading), respiratory infections, cancers and diabetes [1]. In 2019, noncommunicable diseases accounted for 74% of deaths worldwide [1]. Measuring the state of health requires the use of different levels of measurements. Health indicators are measures used to evaluate public health system effectiveness and its functioning [2]. Monitoring mortality rates from leading causes, life expectancy and other health indicators is essential to address their causes and adapt health systems to prevent excess mortality and improve populations’ health status [2,3]. 

Healthcare public health (HCPH) is one of the three main pillars of public health, alongside health improvement and protection [4,5]. Timely and effective care can significantly contribute to improving the health of the population. The potential of healthcare to improve the health of the population depends on its availability, funding and resources. The HCPH components include: focus on the population (by assessing the health needs of the population in specific areas, the population that is able to benefit from a given service must be determined); evidence-based activities (independent interpretation of published evidence, available data or other relevant sources of knowledge); making decisions based on evidence and values to ensure equitable access to effective, safe, patient-centered care; providing advice and support in the monitoring and evaluation of observed changes after the implementation of a given service; cooperation with the payer and other system institutions to best meet the health needs of the population; and cross-industry and partnership cooperation, including with the private sector to develop HCPH services [5].

Studies demonstrate that indicators of current health status give representations of ongoing processes within the population, therefore enabling future public health interventions [2]. Health systems integration is identified as a crucial component of health system reform with the goal of improving health outcomes [6,7]. 

In the study by Tejada et al. based on data from 127 countries, the correlation between macroeconomic indicators, public health expenditure and child mortality rates was found [8]. In the study by Owusu et al., where the data from 177 countries were analyzed, the authors achieved similar results: increasing public health expenditure significantly improves maternal and infant mortality rates worldwide, especially in lower income countries [9]. 

The aim of this study is to present the dependencies of selected health care indicators and health outcomes as an introduction to further analysis of the relationship between systemic and organizational health care factors and health outcomes for the better planning of health care public health services.

## 2. Materials and Methods

In August 2022, a literature review was conducted in order to select the most relevant health indicators. The following queries and keywords were used: “public health” AND “indicators” AND “mortality” AND “disease” AND “financing” AND ‘rates’ AND ‘economy’. The PubMed database was used and search for the grey literature was also performed. The analyses included data from the Organization for Economic Cooperation and Development (OECD) presented in the statistics and the Health at a Glance 2021 report and data collected as part of the preparation of the Financing Global Health 2020 report by the Institute for Health Metrics and Evaluation for reporting countries (Czech Republic, Luxembourg, Norway, Greece, Sweden, Denmark, France, Canada, Australia, Lithuania, Switzerland, Latvia, Spain, Iceland, U.S.A., Canada, Hungary, Estonia, Germany, Austria, Slovenia, Japan Portugal, Ireland, Belgium, The Netherlands, Slovakia, Turkey, Russia, Italy, Finland, UK and Poland) [10]. High-income countries were chosen based on the diversity of the functioning healthcare model and data availability for the selected indicators. For those countries, indicators of access to public health services, financing of the healthcare systems and population health were obtained. 

Then, an analysis of the correlation between the values of the selected healthcare public health indicators and the values of the population’s health indicators was performed using Pearson’s r correlation. The strength of the correlation was interpreted according to the guidelines presented by Mukaka M.M. [11]. The results of the analysis made it possible to demonstrate the correlation between the analyzed indicators and the health result. The selected indicators of the health status of the population (such as life expectancy, deaths from preventable causes and excess deaths) were adopted as the health outcome resulting from the functioning of the health care system.

## 3. Results

Table 1 presents the selected indicators by country and Table 2 shows the selected indicators of the health of the populations in the high-income countries chosen for analysis.

Table 3 shows the Pearson’s r correlation coefficients between selected indicators of the healthcare system functioning evaluation and the indicators of the health of the population. Statistically significant correlations are shown in bold.

The percentage of services financed under general health insurance correlated positively with life expectancy (r = 0.527; *p* < 0.01) and negatively with the number of excess deaths (r = −0.471; *p* < 0.01), the number of deaths from preventable causes (r = −0.405; *p* < 0.05) and the number of deaths from treatable causes (r = −0.442; *p* < 0.01).

Hospital services financed under general health insurance, expressed as a percent-age, correlated positively with life expectancy (r = 0.300; *p* < 0.05) and negatively with the number of excess deaths (r = −0.335; *p* < 0.05).

The above analyses showed that the percentage of expenditure on drugs and medical devices in household budgets correlated negatively with life expectancy (r = −0.665; *p* < 0.01) and with the number of excess deaths (r = 0.366, *p* < 0.05), the number of deaths from preventable causes (r = 0.535; *p* < 0.01) and the number of deaths from treatable causes (r = 0.595; *p* < 0.01). 

A positive correlation was found between the percentage of expenditure on outpatient services in home budgets and life expectancy (r = 0.403; *p* < 0.05) and a negative correlation was found with deaths from treatable causes (r = −0.318; *p* < 0.05).

The percentage of private expenditure on long-term care in home budgets correlated positively with life expectancy (r = 0.482; *p* < 0.01) and negatively with the number of deaths from preventable causes (r = −0.421; *p* < 0.05) and with the number of deaths from treatable causes (r = −0.449; *p* < 0.01).

The number of doctors per 1000 people correlated positively with life expectancy (r = 0.581; *p* < 0.01). It was also shown that more frequent medical consultations led to a re-duction in the number of excess deaths (r = −0.282; *p* < 0.05).

The number of modern diagnostic imaging devices correlated positively with life expectancy (r = 0.433; *p* < 0.01) and negatively with the number of excess deaths (r = −0.348; *p* < 0.05) and the number of deaths from treatable causes (r = −0.365; *p* < 0.05).

The number of nurses per 1000 people correlated positively with life expectancy (r = 0.620; *p* < 0.01) and negatively with the number of excess deaths (r = −0.522; *p* < 0.01), the number of deaths from preventable causes (r = −0.400; *p* < 0.01) and the number of deaths from treatable causes (r = −0.593; *p* < 0.01).

Statistically significant mean positive correlations were found between all public health system financing ratios and life expectancy (r = 0.670, *p* < 0.01, SD = 4.24). The greater the value and share of public expenditure in healthcare expenditure, the longer the expected quality of life. There were also statistically significant negative correlations between all public health funding rates and the number of excess deaths (r = −0.511, *p* < 0.01). 

The negative correlation between public health expenditure per capita and excess deaths was moderate (r = −0.511, *p* < 0.01). The negative correlations between the share of public health expenditure in total expenditure and public health expenditure as part of GDP and the number of excess deaths (r = −0.433, r = −0.404, *p* < 0.01) and between all public health funding rates and the myocardial infarction mortality rate were low (r = −0.426, *p* < 0.01; r = −0.314, *p* < 0.05; r = −0.436, *p* < 0.01, M = 6.65). The greater the value and share of public expenditure in healthcare expenditure, the lower the number of deaths.

The value of public expenditure on health care per capita and public expenditure on health care as part of gross product also negatively correlated with the number of deaths from causes that could have been avoided and the number of deaths from causes that could be treated. 

The negative correlations between the value of public health expenditure per person and the number of deaths from causes that could have been avoided (r = −0.536, *p* < 0.01) and the number of deaths from causes that could be treated were moderate (r = −0.685, *p* < 0.01). The negative correlation between public health expenditure as a part of GDP and the number of deaths from treatable causes was also average (r = −0.595, *p* < 0.01, M = 73.16) (Table 4).

## 4. Discussion

Expenditure on healthcare constitutes an important indicator in the evaluation of the healthcare system functioning. The results of the authors’ study show a positive correlation between the percentage of services financed from public funds and life expectancy. According to the research, an increase in funds for healthcare services translates into a better health result and a longer life in health. According to the OECD analysis, an average 10% increase in health expenditure translates into an increase in life expectancy by 3.5 months [10]. A study conducted by Panahi et al. also indicated the positive impact of increased healthcare expenditure on life expectancy [12]. The greater share of publicly financed services also showed a negative correlation with deaths. The death rate decreases with the increase in the share of services financed under general health insurance. The results of the study conducted by Novignon et al. also indicated a negative correlation between expenditure on health services and deaths [13].

The analysis carried out by the authors showed that a high number of doctors and nurses per inhabitant had a positive effect on the health indicators. This position was also confirmed in the study conducted by Pacakova et al. [14]. In turn, in the study conducted by Winkelmann et al., it was emphasized that the differentiation in regional access to doctors, nurses and midwives in European countries is significant [15].

The results of the authors’ study indicate a negative correlation between private expenditure on drugs and medical devices and life expectancy, as well as a positive correlation with deaths. According to OECD data, private expenditure on drugs and pharmaceuticals is the largest group of costs incurred by patients, more than twice as high as private expenditure on medical services. The research indicates a negative impact of the share of out-of-pocket expenditure on health on the economic situation of the population. According to analyses by World Health Statistics, private expenditure on drugs and medical devices constitutes a significant burden on household budgets. This often forces patients to choose between buying drugs or covering the cost of living. Discontinuation of treatment with pharmaceuticals for economic reasons increases the mortality rate and shortens life expectancy. An increased share of expenditure on drugs and pharmaceuticals is observed in the group of elderly people and is often associated with multi-morbidity in people over 60 years of age [16]. Cardiovascular diseases constitute an example, as they are the leading cause of death among non-communicable diseases in the world. Most of the diagnoses in this group are chronic diseases that require constant treatment using pharmaceuticals. Wang et al., indicated that private expenditure on drugs in cardiovascular diseases is the dominant group of OOP costs burdening home budgets [17]. Economically disadvantaged patients are at risk of discontinuing treatment for financial reasons, which may translate into a higher mortality rate due to cardiovascular diseases [18]. 

### Limitations

The authors included data from high-income countries alone, which could be considered as a limitation. Healthcare indicators were chosen by authors based on the relevance; however, this might be considered as limitation, since there are many public health indicators that are considered crucial. Additionally, it is worth mentioning that only linear correlation analysis between the selected indicators was performed. This article is an introduction to an in-depth analysis. Its purpose is not to propose a system for monitoring indicators for the evaluation of the health system functioning.

## 5. Conclusions

Based on the conducted analyses, it was found that life expectancy is positively correlated with access to general health insurance and public expenditure on healthcare, which indicates the need for all countries to strive to provide their citizens with broad access to healthcare services. The analyses also confirmed that a higher number of doctors and nurses has a positive effect on health results in society and contributes to a reduction in the number of deaths, while insufficient investment in public health measures, including HCPH, increases the incidence and burden of preventable diseases. Limiting the inadequate use of funds, as well as focusing on preventive measures, can contribute to improving the efficiency of expenditure in this sector. 

Due to the changing health care system and the evolution of public health areas, it is important to introduce changes to the organization of the system in order to best match health needs. Expenditure on health care is constantly growing; it is a heavy burden for the state budget, and other systemic problems, such as shortages of medical staff, also arise. Healthcare financing requires in-depth analysis and reorganization. The current macroeconomic challenges started in 2020 by the COVID-19 pandemic are also affecting the health care system. This applies to state budgets and private budgets. Increasing financial challenges translate into limitations in access to health services and translate into a worse health result. A significant share of private expenditure on drugs in the structure of household expenditure is an undesirable phenomenon that may lead to the exclusion of people with a worse socio-economic situation. 

The above analyses indicate the existence of significant systemic problems in developed countries, which should be addressed in the face of growing challenges in these countries, such as the aging of the population, increasing current expenditure on health services and problems with the replacement of medical staff.

## Figures and Tables

**Table 1:** Selected indicators of the health care system in OECD countries (last available data).

Country	% Services Financed under General Health Insurance	% Hospital Services Financed under General Health Insurance	% Private Expenditure on Drugs and Medical Devices in Household Budgets	Number of Medical Consultations per 1 Person	Number of Consultations per 1 Doctor	Number of Imaging Diagnostic Devices—PET, MRI and CT per 1 mln of Population	Number of PET, MRI and CT Examinations per 1000 of Population	Number of Doctors per 1000 People	Number of Nurses per 1000 People
Norway	86	99	36	4.4	886	44.9	205	5.0	17.88
Germany	85	97	36	9.8	2230	71.4	292	4.4	13.95
France	84	96	26	5.9	1880	36.0	332	3.2	11.07
Denmark	83	90	39	4.0	977	48.9	285	4.2	10.10
The Netherlands	83	91	39	8.8	2369	33.2	177	3.7	10.69
Slovakia	80	86	65	11.1	3112	28.8	236	3.6	5.74
Belgium	77	77	28	7.3	2308	38.5	313	3.2	11.07
Austria	75	88	36	6.6	1241	56.4	349	5.3	10.37
Estonia	74	98	40	5.5	1585	35.4	188	3.5	6.24
Italy	74	96	38	10.4	2567	70.2	178	4.1	6.16
Poland	72	93	69	7.7	3197	28.3	144	2.4	5.10
Spain	71	88	53	7.3	2369	38.6	228	4.4	5.89
Canada	70	91	47	6.6	2412	26.2	210	2.7	9.98
Hungary	68	90	50	10.7	3063	15.3	242	3.5	6.62
Australia	67	62	48	7.3	1908	88.3	196	3.8	12.22
Switzerland	67	84	18	4.3	1001	67.6	208	4.4	17.96
Litwa	66	91	52	9.5	2079	41.2	200	4.6	7.74
Latvia	61	87	49	6.1	1867	53.8	254	3.3	4.39
Greece	60	65	37	3.2	519	75.7	285	6.2	3.38
Czechia	82	94	57	8.2	2016	28.4	180	4.1	8.56
Luxembourg	85	93	38	5.5	1910	32.3	319	3.0	11.72
Sweden	85	99	45	2.6	625	45.1	-	4.3	10.85
Lithuania	66	91	52	9.5	2079	41.2	200	4.6	7.74
Iceland	83	99	42	-	-	69.3	340	3.9	15.36
United States	-	-	-	-	-	90.8	413	2.6	11.98
Portugal	61	79	32	-	-	29.6	270	5.3	7.08
Ireland	75	72	29	5.8	1749	38.2	-	3.9	12.88
Turkey	-	-	-	9.8	5033	27.3	-	2.0	2.40
Russia	61	74	-	9.9	2381	19.0	-	4.2	8.48
Finland	78	94	41	4.4	-	48.4	121	3.2	14.26
United Kingdom	79	93	43	-	-	16.5	175	3.0	8.20
Slovenia	73	87	47	6.7	2054	32.1	164	3.3	10.28
Japan	84	92	54	12.5	5011	171.3	-	2.5	11.76

- data unavailable.

**Table 2:** Selected demographic indicators in OECD countries (last available data).

Country	Life Expectancy	Excess Deaths (Deaths in 2020—Compared to Average in 2015–2019)	Deaths from Preventable Causes per 100,000 People (Age-Standardized Indicator)	Deaths from Treatable Causes per 100,000 People (Age-Standardized Indicator)
Norway	83.0	−1	98	47
Germany	81.4	7	113	62
France	82.9	11	105	48
Denmark	81.5	2	112	55
The Netherlands	82.2	12	96	49
Slovakia	77.8	12	193	129
Belgium	82.1	15	119	54
Austria	82.0	11	115	55
Estonia	78.8	5	183	98
Italy	83.6	17	84	52
Poland	78.0	22	169	99
Spain	83.9	18	90	51
Canada	82.1	9	116	56
Hungary	76.4	10	243	131
Australia	83.0	−3	93	46
Switzerland	84.0	12	83	39
Lithuania	76.4	14	226	138
Latvia	75.5	5	256	149
Greece	81.7	11	110	69
Czechia	79.3	17	144	90
Luxembourg	82.7	5	41	56
Sweden	83.2	5	91	49
Lithuania	76.4	14	226	138
Iceland	83.2	-	81	45
United States	78.9	20	177	88
Portugal	81.8	14	109	64
Ireland	82.8	-	107	65
Turkey	78.6	-	116	100
Russia	73.2	-	-	-
Finland	82.1	4	122	54
United Kingdom	81.4	13	119	69
Slovenia	81.6	20	131	54
Japan	84.4	-	83	47

- data unavailable.

**Table 3:** Correlation coefficients between the indicators of access to health services and the indicators of the health of the population.

Indicators of the Healthcare Public Health Functioning Evaluation	Selected Indicators of the Health of the Population			
	Life Expectancy	Excess Deaths	Deaths from Preventable Causes	Deaths from Treatable Causes
% services financed under general health insurance	0.527 **	−0.471 **	−0.405 *	−0.442 **
% hospital services financed under general health insurance	0.300 *	−0.335 *	−0.086	−0.118
% private expenditure on drugs and medical devices in household budgets	−0.665 **	0.366 *	0.535 **	0.595 **
Number of medical consultations per 1 person	−0.001	−0.186	0.038	−0.020
Number of consultations per 1 doctor	0.029	−0.133	−0.020	−0.022
Number of doctors per 1000 people	0.581 **	−0.282 *	−0.143	−0.226
Number of nurses per 1000 people	0.620 **	−0.522 **	−0.400 **	−0.593 **
Number of imaging diagnostic devices—PET, MRI and CT	0.433 **	−0.348 *	−0.248	−0.365 *
Number of PET, MRI and CT examinations	0.102	−0.176	−0.081	−0.112

* *p* < 0.05; ** *p* < 0.01; analyses were conducted for countries with available data for selected indicator.

**Table 4:** Correlation coefficients between the public health system funding ratios and population health indicators.

Selected Indicators of the Health of the Population			Financing of Public Healthcare Systems		
	M	SD	Value of Public Expenditure on Health Care per Capita	The Share of Public Expenditure on Health Care in Total Expenditure	Public Expenditure on Health Care as Part of GDP
Life expectancy	79.76	4.24	0.670 **	0.527 **	0.635 **
Excess deaths	11.28	10.22	−0.511 **	−0.433 **	−0.404 **
Deaths from preventable causes	126.00	48.73	−0.536 **	−0.249	−0.378 *
Deaths from treatable causes	73.16	31.54	−0.685 **	−0.227	−0.595 **

* *p* < 0.05; ** *p* < 0.01, M—mean, SD—standard deviation; analyses were conducted for countries with available data.

## Data Availability

Data available at authors.
